# A longitudinal study of environmental tobacco smoke exposure in children: Parental self reports versus age dependent biomarkers

**DOI:** 10.1186/1471-2458-8-47

**Published:** 2008-02-06

**Authors:** Carme Puig, Oscar Garcia-Algar, Toni Monleon, Roberta Pacifici, Piergiorgio Zuccaro, Jordi Sunyer, Cecilia Figueroa, Simona Pichini, Oriol Vall

**Affiliations:** 1 Environmental and Pediatric Research Unit (URIE), Pediatric Department, Hospital del Mar, Barcelona, Spain; 2 Department of Pediatrics, Obstetrics and Gynecology, and Preventive Medicine, Universitat Autònoma de Barcelona, Barcelona, Spain; 3 Department of Statistics, Universitat de Barcelona, Barcelona, Spain; 4 Department of Therapeutic Research and Medicines Evaluation, Istituto Superiore di Sanità, Rome, Italy; 5 Center for Research in Environmental Epidemiology (CREAL), Institut Municipal d'Investigació Mèdica (IMIM), Barcelona, Spain

## Abstract

**Background:**

Awareness of the negative effects of smoking on children's health prompted a decrease in the self-reporting of parental tobacco use in periodic surveys from most industrialized countries. Our aim is to assess changes between ETS exposure at the end of pregnancy and at 4 years of age determined by the parents' self-report and measurement of cotinine in age related biological matrices.

**Methods:**

The prospective birth cohort included 487 infants from Barcelona city (Spain). Mothers were asked about maternal and household smoking habit. Cord serum and children's urinary cotinine were analyzed in duplicate using a double antibody radioimmunoassay.

**Results:**

At 4 years of age, the median urinary cotinine level in children increased 1.4 or 3.5 times when father or mother smoked, respectively. Cotinine levels in children's urine statistically differentiated children from smoking mothers (Geometric Mean (GM) 19.7 ng/ml; 95% CI 16.83–23.01) and exposed homes (GM 7.1 ng/ml; 95% CI 5.61–8.99) compared with non-exposed homes (GM 4.5 ng/ml; 95% CI 3.71–5.48). Maternal self-reported ETS exposure in homes declined in the four year span between the two time periods from 42.2% to 31.0% (p < 0.01). Nevertheless, most of the children considered non-exposed by their mothers had detectable levels of cotinine above 1 ng/mL in their urine.

**Conclusion:**

We concluded that cotinine levels determined in cord blood and urine, respectively, were useful for categorizing the children exposed to smoking and showed that a certain increase in ETS exposure during the 4-year follow-up period occurred.

## Background

Involuntary exposure of preschool children to environmental tobacco smoke (ETS) in home where they spend most of their time is an important and preventable cause of morbidity. Evidence from meta-analyses and systematic reviews shows that ETS increases the risk for sudden infant death syndrome, middle ear disease, respiratory tract infections, and wheezing during childhood [1-3]. Furthermore, ETS exposure of young children has been associated with increase in carcinogen-protein adducts [4], which in turn may be considered as a potential risk of cancer later in life [5,6].

In the last decades, awareness of the negative effects of smoking on maternal and fetal health and the benefits of quitting smoking prompted a decrease in the proportion of smoking mothers in most industrialized countries. Maternal self-reported smoking in periodic population-based surveys revealed a decreasing trend or cessation of cigarette consumption during pregnancy [7-9] and during early life of their children [10,11]. In all these epidemiological studies, parental tobacco self-reporting has been the method of determining ETS exposure at home. According to the questionnaire, passive exposure from other sources beside home, such as bars, restaurants, etc. did not usually occur in children.

Although standardized questionnaires are widely used in indicating the smoking data at home, the frequency of underreporting is high, and the addition of an objective biological marker results in a more accurate estimate of ETS exposure in children [12].

Cotinine is a major metabolite of nicotine and is considered the best biomarker for nicotine exposure. Cotinine can be found in many biological matrices, such as blood, saliva, urine, meconium, and hair. Being a non-invasive test, the urinary cotinine measurement is among the most frequently used methods. Few cross-sectional studies have quantified ETS exposure in newborns [13] and children aged 4 to 16 years [14-17] using urinary cotinine levels. As far as we know, no longitudinal study has been conducted to assess ETS exposure in the same cohort at birth and at a preschool age by testing for urine cotinine.

Our aim wasto assess changes between ETS exposure at the end of pregnancy and at 4 years of age in a prospective birth cohort. We documented in this cohort that cord serum cotinine appeared to be the most adequate biomarker of fetal exposure to smoking at the end of the pregnancy [18]. In the same article (18), we reported a good correlation between cotinine concentrations in cord blood and neonatal urine. In the 4 year olds, the level of ETS exposure was determined by the parent's self-report and measurement of urinary cotinine, the most widely used biomarker for ETS in children.

## Methods

### Study design and participants

The included infants were gathered from the Barcelona cohort taking part in the Asthma Multicenter Infants Cohort Study (AMICS). The AMICS study is an ongoing multicenter observational study with the participation of different cohorts from Ashford (UK), Barcelona, Menorca (Spain), and Munich (Germany), the objective of which is to assess pre- and postnatal effects of different health and environmental factors on the inception of atopy and asthma. The Barcelona cohort included 487 infants from consecutive pregnant mothers attended at the antenatal visit in Hospital del Mar, Barcelona (Spain) between January 1996 and October 1998 who completed the questionnaire. Women were fully informed of the purpose of the study, the design of which included scheduled visits for both mothers and offsprings at fixed time points after delivery. Participants were invited to join the study if they anticipated living in the city during the forthcoming 6-year study period and had a telephone number. The study protocol was approved by the institutional review board and mothers gave written informed consent.

### Demographic and neonatal characteristics

At the first antenatal care visit to the hospital, usually during the third trimester of pregnancy, an exhaustive questionnaire was completed including maternal age, parental birth country and sociodemographic characteristics. Social class was defined by paternal occupation using the UK Registrar General's 1990 classification.

General information on delivery (gestational age, gender, weight and length and head circumference at birth) was recorded from medical records.

### Maternal self reported tobacco exposure characteristics

Mothers were asked at both antenatal and the child's 4 year old visit about maternal, household smoking habit characteristics and the children's passive exposure from other sources beside home. Three categories of mothers were defined: non exposed-non smoker (NENS), exposed-non smoker (ENS), smoker (S). If they were daily smokers, they were asked the average number of cigarettes currently smoked per day and the brand of cigarette. Regarding exposure to ETS, non-smoking and smoking mothers were asked if they were regularly exposed to ETS, where and by whom (husband or other people in the family and/or at work), the average number of cigarettes smoked per day, the average hours of exposure per day and the brand of cigarettes smoked by these people.

Tobacco consumption by nicotine daily intake (NDI) and exposure to ETS by daily exposure to nicotine (DEN) were calculated as milligrams of nicotine NDI = (mg nicotine/cigarette) * (number of smoked cigarettes/day); $\text{DEN}=\sum_{\text{smokers}}{\text{[NDI}}_{\text{smokers}}*\text{(hours spent with the smoker / 24 hours)]}$[19,20].

### Sample collection and chemical analysis

Cord blood (n = 415, 85.2%) was obtained at delivery, immediately centrifuged and the cord serum was collected. Children's urine samples (n = 306, 62.8%) were collected at the age of 4 years. Urine samples were collected at a consistent time, in the morning, following the visit protocol. All samples were stored at -80°C until analysis.

Cord serum and urinary cotinine were analyzed in duplicate using a double antibody radioimmunoassay according to a method described previously [21]. The limit of detection was 0.2 ng/mL. We discontinued creatinine analysis in our study samples due to the low reliability of creatinine analysis in thawed samples as reported in our previous study [18] since creatinine precipitates as small crystals difficult to redissolve. Furthermore, other authors [22] found that creatinine-corrected urine cotinine concentrations showed less correlation with parental smoking history than did the uncorrected values.

Children exposed to ETS were stratified as non exposed (0–1 ng/ml), and low (1–14 ng/ml), medium (14–50 ng/ml), highly (50–100 ng/ml), and very highly (>100 ng/ml) exposed according to cord blood cotinine level groups used in a previous article [18]. In the absence of established cut-offs for urinary cotinine to differentiate levels of ETS exposure in childhood, it was decided to apply the same stratification criteria as used for cord blood. This choice was made considering that urinary cotinine in 4 years only reflects second-hand smoke (and thus only a passive smoking) while cord blood cotinine accounts not only for second hand smoke but also for maternal active smoke directly in contact with the fetus. Furthermore, this choice was supported by the fact that the half life of cotinine does not vary during childhood [23].

The subgroup of 246 (50.5%) children with full data (questionnaire and both biomarkers:cord blood cotinine at birth and urine cotinine at the age of 4 years) was used to explore changes in the prevalence of ETS exposure between the two time points.

### Statistical analysis

We compared sociodemographic and clinical data of mothers and their children using analysis of variance (ANOVA) statistics for continuous variables and chi-square tests for categorical variables and Fisher's exact test in the case of small proportions.

To assess the relationship between maternal self-reported smoking habits and cotinine levels in children's urine at the age of 4 years, cotinine concentrations were log transformed to fit a normal distribution and Pearson coefficient (r) was calculated. Moreover, we used median and geometric means as average for evaluating a dose-response relationship between self reported smoking habits (as the standard) and cotinine levels; groups of DEN in non-smoker mothers and groups of NDI in smoking mothers were stratified according to tertiles. Furthermore, to compare the four year old's urinary cotinine concentrations among children from NENS, ENS, and S, adjusting for potential confounders such as maternal age and sex of the child, a multiple linear regression analysis was conducted.

To assess the relationship between cotinine concentrations in the umbilical cord serum and the 4 year olds' urine samples, cotinine concentrations were log transformed to fit a normal distribution and gamma coefficient (γ) correlation for ordinal variables was calculated.

Statistical significance was set at p < 0.05 and all analyses were performed using SPSS, version 11 (SPSS Inc, Chicago, Illinois, USA).

## Results

Table 1 shows the characteristics of the study population at the two time periods of follow-up. Socio-economic status measured by paternal job had a broad distribution. The maternal ethnic distribution between Spanish (74.5%) and non-Spanish (25.5%) reflected the demography of population attending the Hospital del Mar. In the 4 year interval of follow-up, a significant percentage of non-Spanish mothers could not be contacted for various reasons (moved, telephone number changed, etc), but there were no statistically significant differences in neonatal environmental tobacco exposure between the retained group and the group which was lost in the 4 years span.

**Table 1:** Characteristics of the study population at delivery and at 4 years of age follow-up

	**At delivery N = 415**	**4 year N = 306**	p-value*
**Parents:**			

Maternal age at birth, mean (SD)	29.1 (5.4)	29.8 (5.3)	NS
Father's social class, (%)			
Professional	6.2	7.0	NS
Managerial and technical	10.7	10.9	NS
Skilled (non-manual)	40.9	38.5	NS
Skilled (manual)	22.7	24.1	NS
Partly skilled	16.9	16.0	NS
Unskilled	2.6	3.5	NS
Maternal country, Spain (%)	74.5	81.0	0.01

**Neonates:**			

Sex, male (%)	51.1	53.1	NS
Birth weight (g), mean (SD)	3242.0 (481.7)	3180.4 (492.5)	NS
Length (cm), mean (SD)	49.2 (2.1)	49.1 (2.4)	NS
Craneal perimeter (cm), mean (SD)	34.4 (1.5)	34.2 (1.6)	NS
Low weight (<2500 g), (%)	6.3	8.2	NS
Premature (<37 weeks), (%)	5.8	7.8	NS

NS: no significance

Indeed, in the group lost to follow up, the percentage of smoking mothers evaluated by biomarker was 29.5% and in the group retained for the follow up it was 33.6%, p = 0.27. The main cause of loss at follow up was the non-spanish origin of families (included in Table 1), but all the other characteristics are not different in the retained population compared to the original population, at birth. (sex p = 0.25, birth weight p = 0.17, length p = 1, low weight p = 0.62, prematurity p = 0.35).

Cotinine levels in the urine samples were not normally distributed, ranging from 0.20 to 300.90 ng/mL, with the median at 9.60 ng/mL, 25th percentile at 4.37 ng/mL and 75th percentile at 23.35 ng/mL. Cotininelevels in cord blood are described in our previous publication [18].

When parent's smoking behavior was evaluated, urinary cotinine levels increased in parallel with the number of smokers in the household (Table 2).

**Table 2:** Reported parental smoking habit and cotinine levels in children's urine (ng/mL) at 4 years

**Parental smoking status**	**Urinary cotinine at 4 year (ng/mL)**				
	
	**N**	**Median**	**Mean (SD)**	**Range**	**p-value**
At 4 year:					
No smoker	94	4.60	6.50 (6.68)	0.20 – 36.00	-
Only father	64	6.45	10.57 (11.23)	1.10 – 57.40	0.004
Only mother	52	16.30	27.78 (30.58)	2.60 – 170.20	<0.001
Both mother and father	96	19.60	33.06 (41.47)	2.10 – 300.90	<0.001

There was a significant correlation (r = 0.61, p < 0.001) between maternal smoking as expressed by NDI, and urinary cotinine levels in children, while maternal reporting of ETS exposure of their 4 year old offsprings, assessed as DEN, was slightly less correlated (r = 0.56, p < 0.001) with the cotinine levels in the children's urine (Table 3).

**Table 3:** Cotinine levels (ng/mL) in children's urine and maternal self-reported smoking habit at 4 years

**Maternal self-reported smoking habit at 4 years**	**Urinary cotinine in children at 4 years **(ng/mL)			
	**N**	**Median**	**Adjusted GM**	**CI 95%**

**NENS**	102	4.65	4.53	(3.71–5.48)

**ENS**	62	6.45	7.10*	(5.61–8.99)
DEN child (Tertiles)				
≤ 2	39 (62.9%)	5.40	6.38	(4.98–8.18)
2–4	15 (24.2%)	6.50	7.23	(4.83–10.81)
>4	8 (12.9%)	10.80	11.38	(6.55–19.77)

**S**	142	19.15	19.68*	(16.83–23.01)
NDI mother (Tertiles)				
≤ 3.6	15 (10.6%)	9.90	9.75	(6.14–15.49)
3.6–9	48 (33.8%)	13.45	12.45	(9.59–16.14)
>9	79 (55.6%)	28.50	30.41***	(24.89–37.24)

* p-value < 0.05 in relation to NENS

*** p-value < 0.001 in relation to the first tertile of maternal NDI

GM: geometric mean; CI: confidence interval; NENS: non exposed-non smoker; ENS: exposed-non smoker; S: smoker; NDI:nicotine daily intake; DEN: daily exposure to nicotine.

Cotinine levels in children's urine from self-reported smoking mothers (Geometric Mean (GM) 19.7; 95% Confidence Interval (95% CI) 16.83–23.01) and exposed homes (GM 7.1; 95% CI 5.61–8.99) were significantly higher than in children from non-exposed homes (GM 4.5; 95% CI 3.71–5.48).

Maternal smoking habit according to the self-reports showed that the prevalence of active maternal smoking increased in the four year span (from 34.7% to 48.4%, p < 0.001) while ETS exposure in homes declined from 42.2% to 31.0% (p < 0.01) (Table 4). In line with that evidence, the percentage of children not exposed to tobacco smoke decreased significantly, while a significant increase in the biomarker levels was registered only for low exposure to smoking.

**Table 4:** Prevalence of environmental tobacco exposure: maternal report versus biomarker-based at birth and at 4 years of age

	**At delivery N = 415**	**4th year N = 306**	p-value*
**ETS exposure:**			

By questionnaire:			
Active maternal smoking (NDI >0) (%)	144 (34.7)	148 (48.4)	<0.001
Passive exposure(DEN >0) (%)	175 (42.2) (mother DEN)	95 (31.0) (child DEN)	<0.01
By biomarker:	Umbilical cord blood	Child Urine	
0 – 1 ng/mL (%) (no exposure)	45 (10.8)	5 (2.0)	<0.001
1 – 14 ng/mL (%) (low)	228 (55.0)	186 (60.4)	0.02
>14 ng/mL (%) (medium and high)	142 (34.2)	115 (37.6)	0.35

ETS: environmental tobacco smoke; NDI: nicotine daily intake; DEN: daily exposure to nicotine

Furthermore, to explore changes in the prevalence of ETS exposure, the subgroup of 246 children with both biomarkers:cord blood cotinine at birth and urine cotinine at the age of 4 years was categorized in the same groups of ETS exposure at the two time points (γ = 0.77) (Table 5). The prevalence decreased only at the extreme exposures (no exposure and very high exposure) and increased at medium exposure.

**Table 5:** Biomarker based prevalence of tobacco exposure at birth and at 4 years (N = 246), according to the previously established intervals.

**Level of exposure **(ng/mL)	**Cord blood cotinine **n (%)	**Urinary cotinine at 4 years **n (%)
Non exposure <1	25 (10.2)	4 (1.6)
Low exposure 1–14	135 (54.9)	157 (63.8)
Medium exposure 14–50	23 (9.3)	66 (26.8)
High exposure 50–100	21 (8.5)	16 (6.5)
Very high exposure >100	42 (17.1)	3 (1.2)

Chi-square p < 0.001. Gamma Coef 0.770

## Discussion

Results of this study show that measurement of cotinine levels was useful not only for quantifying ETS exposure but also for categorizing the children exposed to smoking, and non-smoking mothers (categorized further in exposed to other smokers, and non-exposed). Furthermore, as a whole, a decrease in the percentage of non exposed children to tobacco smoke and an increase in the percentage of children with a small-medium ETS exposure have been documented during the 4-year follow-up period. Conversely, the very high exposure found at birth (measuring cotinine in cord blood) and coming from the direct feto-placental passage of high amount of tobacco smoke from heavy smoker mothers disappeared.

The moderate linear relationship between results of maternal self-reporting and urinary cotinine in the 4 year olds was useful to distinguish between the presence or absence of smoking mothers, in agreement with other studies [15-17,24]. On the basis of these results, different authors [25,26] have suggested that the usefulness of self-report questionnaires and urinary cotinine levels to assess ETS exposure was similar. However, in our study, in spite of the small numbers in some categories, we identified a total of 98.4% of our preschool population exposed to ETS, with about 8% children with urinary cotinine levels indicative of high and very high exposure to ETS.

According to the questionnaire, passive exposure from other sources beside home, such as bars, restaurants, etc. did not occur in these children (this was one of the questions in the data base of the study). Exposure from other household members or houses of relatives were not significant, according to the questionnaire as well; usually, these children spent their indoor time at home and school. In Spain, although smoking in some public places (eg. bars and some restaurants) is still permitted, the main source of environmental tobacco exposure for children is their home, usually because of parental smoking.

In the present study, the median urinary cotinine level in children living in households where the father smoked was 1.4 times higher and 3.5 times higher when mother smoked compared with children of non-smoking households. Our results are in good agreement with the results of several other studies reporting stronger effect of maternal smoking than paternal smoking on children's ETS exposure [15,27-30]. This maternal influence could be explained by the fact that children spend more time with their mothers at home. Therefore, if we consider that 98.4% of our study population is exposed to ETS and that this is mainly due to maternal smoking, we conclude that the percentage of children living in smoking households in our study is higher than the 38% of preschool children in the USA [31], 50% in the UK [32] and 57% in Northern European countries [33] and only similar to the 72.3% in Turkey [17]. It should be noted that maternal self-reported ETS exposure in homes declined from 42.2% at pregnancy to 31.0% 4 years later. However, most of the children considered non-exposed by their mothers had detectable levels of cotinine above 1 ng/mL in their urine, indicating even more widespread ETS exposure than reflected by maternal self-reporting. It is unlikely that the finding of widespread ETS exposure resulted from deliberately underreported parental tobacco use. Christensen et al. [34] have suggested that the awareness of health risks from ETS exposure could be a bias for assessing objectively the ETS exposure. Therefore, the results of this study support the proposal that parental reports should not be completely trusted when evaluating ETS exposure among non-smoking parents and the use of biomarkers could be a more objective evaluation.

The detectable levels of urinary cotinine for most children reflect more accurately the amount of ETS exposure. Further research is needed to define better the degree of health risks associated with specific values of serum cotinine. It is noteworthy, that in this study, in every matrix, cotinine concentrations are considerably higher than those reported by other authors [13,16,26,29]. Our results suggest that, these children are probably at even higher risk of developing asthma or other deleterious effects of tobacco exposure [35].

Moreover, even though community-level interventions, such as limiting of smoking in public places, may be able to reduce some sources of outdoor exposure, other interventions will be necessary to insure tobacco-smoke free households. Parental education is of paramount importance to avoid ETS exposure.

## Conclusion

The results of this study support the proposal that parental reports should not be completely trusted when evaluating ETS exposure among non-smoking parents and the use of biomarkers could be a more objective evaluation.

Cotinine levels determined in cord blood and urine, respectively, were useful for categorizing the children exposed to smoking and reflected the changes in the degree of exposure at the two time periods. Our results indicate that maternal smoking makes a significant contribution to children's ETS exposure. To our knowledge no other studies have measured ETS exposure in the same pediatric population at two different periods, at birth and at 4 years of age. Children should be more protected from ETS at home. Parents must be strongly advised to quit smoking around their children.

## Competing interests

The author(s) declare that they have no competing interests.

## Authors' contributions

OGA and CP had primary responsibility for study design, protocol development, patient screening, enrollment, outcome assessement, and writing the manuscript.

CF contributed primarily to patient enrollment, and acquisition of data.

JS, PZ and RP, participated in the development of the protocol, and discussion of results.

TM executed statistical analysis of data and participated in results discussion.

SP supervised the design and execution of the study, performed the final data analyses, and contributed to the writing and revising of the manuscript.

OV participated in the design and the coordination of the study, and helped to draft the manuscript.

All the authors read and approved the final manuscript.

## Pre-publication history

The pre-publication history for this paper can be accessed here:


